# An Association Study of the A218C Polymorphism of the Tryptophan Hydroxylase 1 Gene with Eating Disorders in a Korean Population: A Pilot Study

**DOI:** 10.4306/pi.2009.6.1.44

**Published:** 2009-03-31

**Authors:** Youl-Ri Kim, Jong-Min Woo, Si Young Heo, Jeong Hyun Kim, Soo-Jin Lim, Bum-Hee Yu

**Affiliations:** 1 Eating Disorders Clinic, Department of Psychiatry, Seoul Paik Hospital, Inje University School of Medicine, Seoul, Korea.; 2 Stress Research Institute, Inje University, Seoul, Korea.; 3 Nanum Eating Disorders Clinic, Seoul, Korea.; 4 Indang Institute of Molecular Biology, Inje University, Seoul, Korea.; 5 Department of Psychiatry, Samsung Medical Center, Sungkyunkwan University School of Medicine, Seoul, Korea.

**Keywords:** Tryptophan hydroxylase, Eating disorders, Bulimia nervosa, Obsessionality

## Abstract

**Objective:**

We examined the association between the tryptophan hydroxylase 1 (TPH1) gene and eating disorders focusing on obsessionality.

**Methods:**

The sample included 62 women with a lifetime diagnosis of anorexia nervosa (AN) as well as 50 women with a lifetime diagnosis of bulimia nervosa (BN) recruited from specialist clinics for eating disorders and 131 healthy women in Korea. Blood samples were collected from all participants for the TPH1 genotyping. The patients were ad ministered the Korean version of the Eating Disorders Examination and obsessionality was conceptualized using measures of persistence, harm avoidance, and obsessive-compulsive symptoms.

**Results:**

In the case-control comparisons, the frequency of the A/A genotype was increased in the patients with BN, but this difference was not significant after correcting for multiple testing. We found no effect of the TPH A218C polymorphism on obsessionality in the patients with AN or BN.

**Conclusion:**

Although the present findings should be regarded as preliminary because of the small size of our sample, they suggest that the TPH1 gene may contribute to the genetic susceptibility to BN and be associated with the other unexplored traits of bulimic case status.

## Introduction

There is compelling evidence to suggest that eating disorders are marked by a disturbance in the serotonergic system, in which the synthesis of 5-HT is one of the key processes. Furthermore, individuals at risk of developing bulimia nervosa (BN) have trait abnormalities in the regulation of their brain serotonin function that might increase their vulnerability to dieting-induced decreases in plasma tryptophan.^1^ The gene responsible for 5-HT synthesis, the tryptophan hydroxylase gene (TPH), is a plausible candidate for susceptibility to eating disorders. Recently, several separate lines of biological evidence have emerged, which suggest that TPH1 may play an important role in modulating serotonergic function in the brain.^2^-^4^ Moreover, the level of messenger RNA (mRNA) of the TPH1 gene is similar to or higher than that of TPH2 in the brain regions involved in the modulation of both the feeding and emotional aspects of behaviour, such as the cortex, thalamus, hippocampus, amygdala, and hypothalamus areas.^5^ This biological evidence together with a recent genetic association study on BN^6^ point to the involvement of TPH1 in various psychiatric conditions including eating disorders.

In a large multicentre genetic study, obsessionality was selected as an important trait for linkage analysis in eating disorders.^7^ Obsessionality is a salient personality feature of individuals with eating disorders^8^,^9^ and is a heritable trait.^10^ Family studies have reported the increased prevalence of obsessive compulsive disorder and obsessive compulsive personality disorder in relatives of individuals with eating disorders.^11^ Persistence, which is a cognitive component of obsessionality,^12^ is suggested to be a part of the eating disorder endophenotype.^13^ The 5-HT system has been strongly implicated in the regulation of obsessional behavior^14^ or cognitive inflexibility.^15^

Therefore, we investigated whether a genetic variant of the TPH1 gene is associated with eating disorders with particular emphasis on obsessionality. In the absence of any available functional polymorphism in the coding region of the TPH1 gene, the intron 7 A218C polymorphism has been one of the most widely studied polymorphisms in the TPH1 gene and might affect the expression of TPH1.^16^

In the present study, the first aim was to investigate whether the TPH1 A218C polymorphism was associated with eating disorders using a case-control design. Another related objective was to explore the association between the psychopathology of obsessionality and eating disorders and the TPH1 A 218C polymorphism using psychological measurements.

## Methods

### Study population

A total of 243 individuals, comprising women with AN (n=62), women with BN (n=50), and healthy women (n=131), were included in this study. All of the patients were native Koreans, recruited between 2006 and 2008 from two specialist treatment centers for eating disorders in Korea. The patients with eating disorders had a diagnosis of AN or BN according to the Diagnostic and Statistical Manual of Mental Disorders 4th edition (DSM-IV) criteria.^17^ Lifetime diagnoses on the basis of the hierarchical model of diagnosis adapted from a previously reported genetic study^18^ were determined for each patient. The life-time diagnosis was made using selected behavioural items, information regarding the current weight and height, and the current and past menstrual functioning items of the Eating Disorders Examination Questionnaire (EDE-Q)^19^- Korean version. In the individuals with AN, 34% (n=21) reported restricting type and 66% (n=41) reported binge purging type. Of the patients, 42 women with AN and 30 women with BN (67.2%) were administered the Korean version of the Eating Disorders Examination Interview (EDE)^20^ by a psychologist (S-J.L.) and had their behavioural traits of obsessionality assessed. There was no difference between the genotyping only group and the assessment completed group in terms of their demographic and clinical data {age, body mass index (BMI), duration of illness, depression and anxiety}(data available on request).

The healthy controls were recruited by advertisement from among the residents of Korea after careful screening for a current or past history of eating disorders using the EDE-Q adapted to capture episodes of eating disorders in their lifetime and for a personal history of neuropsychiatric disorders. For all participants, the measured weight and height were obtained at the time of assessment, and the highest and lowest ever BMI were determined. This study was approved by the Institutional Review Board of Seoul Paik Hospital. Written informed consent was obtained from all participants.

### Measures

The EDE was used to capture the eating disorder pathology in patients using the restraint, eating concern, weight concern, and shape concern subscales. Obsessionality was conceptualized using traits of persistence and harm avoidance, and obsessive-compulsive symptoms. Persistence and harm avoidance were assessed with the Korean version of the Temperament and Character Inventory (TCI).^21^,^22^ Obsessive-Compulsive symptoms were measured by both a clinicians rating assessment using a composite score for the Yale-Brown Obsessive Compulsive Scale (Y-BOCS)^23^ and self-rating scales using the Korean version of the Maudsley Obsessive-Compulsive Inventory (MOCI).^24^ The level of depression and anxiety in all of the patients was assessed using self-report instruments including the Korean versions of the Beck Depression Inventory (BDI)^25^ and the State and Trait Anxiety Inventory (STAI).^26^

### Genotyping

Genomic DNA was extracted from blood leukocytes using a DNA extraction kit (ABI, Foster City, CA, USA). Genotyping of the TPH polymorphism (A218, rs1800532) was carried out using an ABI PRISM SNaP Shot Multiplex kit and an ABI Prism 3730xl DNA analyzer (ABI, Foster City, CA, USA) according to the manufacturer's recommendations. The primers used for PCR were: sense, 5'-CATGTTCCATGCTCTATATGTGT-3' and antisense, 5'-TGTCTGATTTTTTTCAGTGTTACATT-3'. The PCR conditions were as follows: 10 min at 95℃ for 1 cycle, and 30 cycles each at 95℃ for 30s, 55℃ for 1 min, 72℃ for 1 min followed by 1 cycle at 72℃ for 7 mins. After amplification, the PCR products were treated with 1 unit each of shrimp alkaline phosphatase (Roche) and exonuclease I (USB Corporation) at 37℃ for 60 minutes and 72℃ for 15 minutes. One µL of the polymerase chain reaction (PCR) products was added to a SNaPshot Multiplex Ready reaction mixture containing 0.15 pmols of the genotyping primer of 5'-TTATTAATTGACAACCTATTAGGTG-3' for the primer extension reaction. The results were analyzed using Gene-Scan analysis software (ABI, Foster City, CA, USA).

### Statistical analyses

First, case-control genetic comparisons were designed to evaluate the differences in the genotype and allele frequencies by the chi-square test for the healthy controls and patients (AN or BN). Next, a multivariate analysis of variance (MANOVA) was computed for the patients with AN or BN by integrating the four subscales of EDE or the composite measurements of obsessionality which comprise the Y-BOCS and the MOCI, as well as the two subscales of the TCI, persistence and harm avoidance, and the genotype as an independent variable. The MANOVA was followed by a univariate analysis. p values <0.05 were considered to be indicative of statistical significance, and two-tailed tests were used. A Bonferroni correction was applied if multiple testing was used. All statistical tests were performed with SPSS version 11.0.

## Results

### Clinical characteristics

No difference was found between the groups with respect to age, but the body mass index BMI {wt (kg)/ht (m)^2^}, differed between the groups as expected (Table 1). In the post hoc analysis, the current BMIs of the BN group and controls were similar. The BN group had a higher "highest ever BMI" than the control group. The AN group had the lowest "lowest ever BMI", while that of the BN group was lower than that of the control group. The participants with eating disorders reported moderate levels of depression and anxiety and the BN group reported being more depressive than the AN group.

### Tryptophan hydroxylase 1 A218C polymorphism and eating disorders with case-control comparison

The genotype and allele frequencies of the variants in the participants with AN and BN and controls are shown in Table 2. The TPH1 A218C genotype distribution was in Hardy-Weinberg equilibrium in the patients with eating disorders and healthy participants (χ^2^=2.01, df=2, p=0.16), BN (χ^2^=1.77, df =2, p=0.18) and AN (χ^2^=0.54, df=2, p=0.46). As shown in Table 2, there were no differences in the TPH1 A218C allele/genotype frequency between the healthy controls and the individuals with AN. There was evidence of an association between the A/A genotype and BN, which was not statistically significant after correction for multiple testing.

### Tryptophan hydroxylase 1 A218C polymorphism and psychopathologies for eating disorders and obsessionality in eating disorders

A MANOVA was computed for the patients with eating disorders by integrating the four EDE subscales or the composite measurements for obsessionality. We could not find any effect of genotype on the psychopathologies for eating disorder or obsessionality in either the patients with lifetime AN (EDE; F=1.04, df=8/74, p=0.42; obsessionality; F= 0.99, df=8/74, p=0.46) or those with lifetime BN (EDE; F=1.60, df=8/50, p=0.15; obsessionality; F=0.43, df=8/50, p=0.89). In the subsequent detailed univariate analysis designed to test for genotype effects (Table 3), there were no effects of genotype on the EDE subscales or on any of the measurements for obsessionality.

## Discussion

In this study, a modest association between the A218C genotype of the THP1 gene and BN was found, although the significance was abolished after a correction for multiple testing was applied. This finding shows that the possibility of there being an association between TPH1 and bulimic case status cannot be excluded. There was no significant effect of the A218C genotype of the THP1 gene on the psychopathologies for eating disorders or obsessionality in the analysis focusing on this issue.

The increased prevalence of the AA genotype in BN could be explained by interactions of the THP1 gene with other genes responsible for the serotonergic function in this population. While TPH2 is considered to control 5-HT synthesis in the brain,^27^ recent studies suggest that the TPH1 and TPH2 genes may be coordinately expressed to regulate serotonergic function.^28^,^29^ In addition, Ono et al.^2^ argued that the single nucleotide polymorphisms (SNPs) of the THP1 gene might affect the regulation of the 5-HT_2A_ receptor by producing changes in TPH activity, based on their finding that the AA genotype of the A218C SNP was associated with a lower 5-HT_2A_ receptor density, which is of interest in eating disorders, because it has been implicated in the modulation of feeding^30^,^31^ and women who recovered from BN had reduced 5-HT_2A_ receptor binding potential relative to controls.^14^ Lastly, the A218C SNP might be associated with the turnover of 5-HT via almost complete linkage disequilibrium (LD) with the A779C SNP, whose genotype was reported to be associated with a blunted prolactin response to fenfluramine and low levels of cerebrospinal fluid (CSF) 5-hydroxyindoleacetic acid (5-HIAA),^32^ which in turn is associated with BN.^33^ Our results may reflect the existence of a broken link in the, genetically or functionally, tightly coordinated regulation of the serotonergic function or just one of several malfunctional SNPs that could collectively perturb serotonergic function.

From the finding that there was no significant effect of the A218C genotype of the THP1 gene on the psychopathology for the disorder or obsessionality, it is noteworthy that the A218C polymorphism has an association with other unexplored behavioral traits in this study, such as aggression and anger-related traits^34^-^36^ which are strongly suspected of being personality traits associated with BN.^37^ Therefore, the association between TPH1 and these traits needs to be explored in BN in the future.

We need to consider that the A218C SNP is not the sole determinant of the functionality of the TPH1 gene, because it is in LD with putative functional SNPs in the promoter region, such as A-1067G and G-347T,^16^ as well as other intron SNPs.^36^ As our results show that the A218C SNP alone is not a major genetic risk factor for eating disorders, the analysis of the effect of the haplotypes and, more importantly, of the functionality of each haplotype, on the expression of the THP1 gene is required to clarify the involvement of the THP1 gene in eating disorders.

Our findings support those of previous studies involving Caucasian samples,^6^,^38^ but are somewhat incongruent. In the study of Monteleone et al.,^6^ although no difference was found in the distribution of the TPH1 A218C genotype between the patients with BN and controls, the bulimic women with the AA genotype exhibited more severe binge eating behavior and higher harm avoidance scores. One explanation for the discrepancy between our findings and theirs may be the different criteria used for the phenotype. Considering that between 22% and 37% of women with BN reported a history of AN,^39^ the significant numbers of women with a current diagnosis of BN in their study may be classified into the lifetime binge and purging types of AN in our hierarchical diagnosis. We need to consider a few limitations in this study. The first limitation is the small sample size, which can lead to both type I and type II errors. It is quite possible that we lacked sufficient statistical power to detect relatively small associations. The reduced sample size in the subsidiary analyses focusing on obsessionality resulted in a further reduction of statistical power. Although our sample size is relatively small for a genetic association study, its findings are strengthened by the use of rigorous criteria for lifetime diagnosis to define the phenotype. Thus, our data demonstrate the need for further, large-scale studies in patients with eating disorders. The second limitation is that we did not apply a structured diagnostic interview to the control subjects. However, we do not believe that this could be a confounding factor, as the controls were carefully screened for eating disorders by means of an interview, as well as for a current and past history of psychiatric illnesses, before their participation in this study.

In conclusion, we found a modest increase in the frequency of the AA genotype of the TPH1 gene in the BN group, which supports the hypothesis that the TPH1 gene contributes to predispose individuals to BN.^6^ Based on the findings in this study, we cannot exclude the possibility that unexplored factors might be involved in the association. The results of the present study are informative, but require further analysis of the effect of each haplotype on the expression of the THP1 gene to clarify the latter's involvement in BN in a large-scale population with eating disorders.

## Figures and Tables

**TABLE 1:**
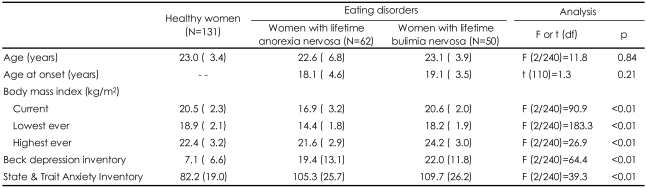
Characteristics of samples

Data were shown as mean (SD)

**TABLE 2:**
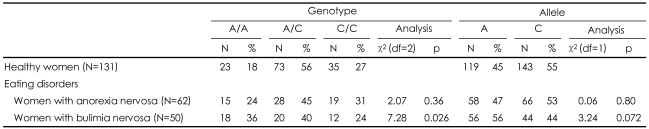
Frequency of genotypes and alleles for the TPH1 A218C gene in healthy women and women with eating disorders

For comparison of eating disorders (anorexia nervosa or bulimia nervosa) patients with healthy controls. Because multiple comparisons were made, the Bonferroni-corrected significance level was set to alpha=0.05/2 (p<0.025)

**TABLE 3:**
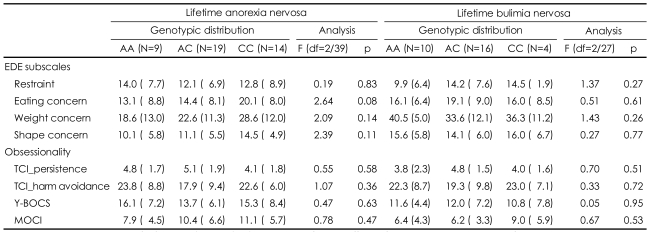
Mean scores for the Eating Disorders Examination Interview or obsessionality as a function of TPH genotype in women with eating disorders

Data were shown as mean (SD). Analysis by univariate ANOVA for the effect of genotype on subscales of the EDE or each measurement for obsessionality. EDE: Eating Disorders Examination Interview, TCI: Temperament And Character Inventory, Y-BOCS: Yale-Brown Obsessive Compulsive Scale, MOCI: Maudsley Obsessive Compulsive Inventory
